# Starvation to Glucose Reprograms Development of Neurovascular Unit in Embryonic Retinal Cells

**DOI:** 10.3389/fcell.2021.726852

**Published:** 2021-11-18

**Authors:** Türküler Özgümüs, Oksana Sulaieva, Ruchi Jain, Isabella Artner, Valeriya Lyssenko

**Affiliations:** ^1^ Department of Clinical Science, Center for Diabetes Research, University of Bergen, Bergen, Norway; ^2^ Medical Laboratory CSD, Kyiv, Ukraine; ^3^ Department of Clinical Sciences, Lund University Diabetes Centre, Skåne University Hospital, Malmö, Sweden

**Keywords:** transcriptomics, retinogenesis, starvation, neurovascular unit, retinopathy, transcriptomic (RNA-seq)

## Abstract

Perinatal exposure to starvation is a risk factor for development of severe retinopathy in adult patients with diabetes. However, the underlying mechanisms are not completely understood. In the present study, we shed light on molecular consequences of exposure to short-time glucose starvation on the transcriptome profile of mouse embryonic retinal cells. We found a profound downregulation of genes regulating development of retinal neurons, which was accompanied by reduced expression of genes encoding for glycolytic enzymes and glutamatergic signaling. At the same time, glial and vascular markers were upregulated, mimicking the diabetes-associated increase of angiogenesis—a hallmark of pathogenic features in diabetic retinopathy. Energy deprivation as a consequence of starvation to glucose seems to be compensated by upregulation of genes involved in fatty acid elongation. Results from the present study demonstrate that short-term glucose deprivation during early fetal life differentially alters expression of metabolism- and function-related genes and could have detrimental and lasting effects on gene expression in the retinal neurons, glial cells, and vascular elements and thus potentially disrupting gene regulatory networks essential for the formation of the retinal neurovascular unit. Abnormal developmental programming during retinogenesis may serve as a trigger of reactive gliosis, accelerated neurodegeneration, and increased vascularization, which may promote development of severe retinopathy in patients with diabetes later in life.

## Introduction

Intrauterine or fetal programming has in the past years been connected to age-related metabolic diseases in adults (Vaiserman and Lushchak, 2019; Hsu and Tain, 2020). Thus, there is striking evidence indicating that 25–63% of diabetes, cardiovascular disease, hypertension, and obesity can be attributed to the perinatal factors and features such as low weight at birth (Tian et al., 2006). Notably, pediatric ophthalmologists were the first to observe that preterm and low-birthweight infants exhibited a lasting abnormal retinal architecture, suggesting a key role of the intrauterine environment for vascular development in the retina and retinal neurovascular unit (RNVU) formation (Swan et al., 2018). Retinopathy is a progressive complication of diabetes with a global prevalence of 35.4% among diabetic patients (Solomon et al., 2017). Proliferative diabetic retinopathy (PDR) is the most severe form, which is characterized by progressive neovascularization leading to severe vision loss and blindness (Wong et al., 2018). Presently, there are only a few treatment alternatives for severe retinopathy targeting vascular pathology such as laser photocoagulation and anti-VEGF therapy (Wong et al., 2018). However, these treatments are not affordable in every country, and the prevalence of PDR is reported to be higher in developing countries as compared to developed countries (Ruta et al., 2013).

In support of the fetal programming hypothesis, recent epidemiological observational studies from the Ukrainian and the Hong Kong Diabetes Registries demonstrated disproportionally elevated risk for severe diabetic retinopathy in offspring to parents exposed to famine (Fedotkina et al., 2021). This evidence indicated that fetal exposure to starvation can be a triggering risk factor for vision-threatening diabetic retinopathy in adults, which might, independently or synergistically with diabetes-related metabolic risk factors, aggravate disease progression (Fedotkina et al., 2021). Understanding mechanisms involved in early retinogenesis during starvation insults, which could increase preponderance to diabetes retinopathy later in life, might aid in discovering fundamental cues for novel treatment strategies. In the present study, we investigated the effects of short-term exposure to glucose starvation on global transcriptome changes of embryonic retinal cells comprising mostly neuronal, glial, and vascular cells.

## Materials and Methods

### Retinal Cell Sample Preparation and Culture, RNA Isolation, and Sequencing

*Isolation and Culture of Retinal Cells*. C57BL/6J mice were purchased from Charles River. The retinas were isolated from E18.5 mouse embryos and digested with 0.05% trypsin (ready-made, Gibco) for 15 min at 37°C. The digestion was terminated by adding Dulbecco’s modified Eagle’s medium (Gibco) supplemented with 25 mM sodium bicarbonate (Gibco), 25 mM HEPES (Gibco), 10% fetal bovine serum (v/v, HyClone), and 1% penicillin and streptomycin solution (v/v, Gibco). The cell suspension was filtered through a 70-μM filter and centrifuged at 1,300 rpm for 5 min, resuspended in the medium, and centrifuged. This was repeated twice, and the cells were plated on poly-l-lysine-coated plates at a density of 2.0 × 10^6^ cells/cm^2^. Next day, the cells were washed two times with phosphate-buffered saline (PBS) and starved for glucose in the Neurobasal medium supplemented with B27 supplement lacking insulin, with 0.06 g/l-glutamine, 1% penicillin–streptomycin (v/v, Gibco), and 11 mM HEPES for 6 h. The cells were further cultured for 6 days in the normal glucose medium (complete Neurobasal medium). The cells were harvested after 6 days of culturing for RNA isolation to obtain information on the long-term effects of starvation exposure.

Total RNA was isolated by using the miRNeasy micro kit (Qiagen), and reverse transcription was done by using the RevertAid first-strand cDNA synthesis kit (Thermo Fisher). RNA extracts were isolated by using TruSeq Stranded Total RNA with Ribo-Zero. Sequencing was done paired-end on the Illumina NextSeq 500.

The study was approved by the local ethics committee (Regional Ethics Review Board, Lund, Sweden, 2018-579, 2016/891), and the experiments were performed in compliance with the ARRIVE guidelines (Kilkenny et al., 2010).

### Processing and Analysis of RNAseq Data

A total of 12 samples were sequenced (*n* = 6). One of the control samples was excluded from further analysis since it was not clustering with other control samples on the principal component analysis (PCA) biplot. The quality of paired-end RNAseq files was checked with MultiQC v1.0 by using fastq files. A Phred score greater than 30 was achieved for each sequencing position for all samples. There was no adapter contamination shown in QC (adapter contamination <0.1%), so trimming was not applied in order not to lose information. The alignment of transcripts was done by using Kallisto v0.43.1 (Bray et al., 2016) with the GRCh38.p10 reference assembly with the Ensembl *Mus musculus* v90 annotation as the reference transcriptome (Yates et al., 2016).

On average, we obtained 17.2 ± 6.7 (mean ± SD) million paired-end reads mapped to the mouse genome. After excluding lowly expressed genes (expressed in less than 20% of the samples), a total of 22,978 genes were used in the downstream analyses. The downstream analysis from this level was done by using the R statistical environment (www.r-project.org) and R Studio (www.rstudio.com). The estimated count values of the transcripts given as Kallisto output were converted to gene-level expression values by using Tximport v1.6.04 (Soneson et al., 2015). The genes that had low counts were excluded from the expression matrix with the inclusion criteria of a CPM higher than 0.5 for at least two of the samples.

PCA was applied to explore the differences between libraries. A PCA biplot and sample heatmap were prepared by using a regularized log transformation (rlog) function in the DESeq2 package (Love et al., 2014). Based on exploratory data analysis, one of the control samples was excluded from further analysis since it stood as an outlier on the PCA biplot showing the first two principal components based on gene expression counts. The PCA biplot showing the first two principal components that explain 64% of variation across the samples and the sample clustering based on the gene expression are presented in Supplementary Figure S1. The starved and control samples were separated along the first principal component axis, which shows that the gene expression was a determinant for the separation of the conditions and that most of the explained variance is primarily due to the difference between two conditions. The heatmap shows the clustering of the samples based on Euclidean distances between the samples. The distances are smaller within the conditions; hence, the samples are clustered based on the conditions.

The differential expression analysis was done with using edgeR v3.20.73 (Robinson et al., 2010; McCarthy et al., 2012). The raw count values were provided to edgeR since the tool handles the normalizations for sequencing depth, gene length, and RNA composition of the libraries (TMM normalization). Condition is used as a predictor of gene expression in a quasi-likelihood negative binomial generalized log-linear model (glmQLFit function). Empirical Bayes quasi-likelihood *F*-tests were used to assess the differential expression (glmQLFTest function). Batch was not included as a covariate in the final analysis since there was no obvious batch effect seen in the data exploration, and above 90% of the differentially expressed genes remained significant when the batch was added in the formula. Sex adjustment was not done via adding sex as an explicit covariate, but a robust algorithm was used to down-weight sex-linked genes (Phipson et al., 2016). False discovery rate (FDR) correction was done by using the Benjamini–Hochberg method. The gene was considered differentially expressed in case of FDR <0.05.

### Cell Marker Selection

Marker genes for different cell types were selected based on literature (Fruttiger, 2002; Haverkamp et al., 2003; Casini et al., 2006; Ponomarev et al., 2006, 40; Kim et al., 2008; Johansson et al., 2010; Sanes and Zipursky, 2010; Winkler et al., 2010; Armulik et al., 2011; Kay et al., 2011; de Melo et al., 2011; Bassett et al., 2012; de Melo et al., 2012; Wu et al., 2013; Seung and Sümbül, 2014; Vlasits et al., 2014; Zhao et al., 2014; Darmanis et al., 2015; Karlstetter et al., 2015; Macosko et al., 2015; Maddox et al., 2015; Sanes and Masland, 2015; Zeisel et al., 2015; Boije et al., 2016; Gill et al., 2016; Shekhar et al., 2016; Struebing et al., 2016; Tasic et al., 2016; Vecino et al., 2016; Yu et al., 2016; Hoshino et al., 2017; Lee et al., 2017; Welby et al., 2017; Zeng and Sanes, 2017; Keeley and Reese, 2018; McDowell et al., 2018; Rheaume et al., 2018; Roesch et al., 2008). Genes were considered as marker genes of a relevant cell type upon demonstrating cell-specific expression in at least two separate publications. Cell type-specific expression has been confirmed using immunostaining or single-cell analysis in previous studies. Hence, many of them are overlapping between cell types, especially the ones that belong to the same cell classification as neuronal, glial, or vascular.

### Gene Ontology and Pathway Enrichment Analyses

GO and pathway enrichments were done by using goana and kegga functions in edgeR. The genes in the relevant GO terms were extracted from the main page of gene ontology (www.geneontology.org) and KEGG (genome.jp) databases (Ashburner et al., 2000; Kanehisa and Goto, 2000; Mi et al., 2017; The Gene Ontology Consortium, 2017). The following packages were used for the creation of figures: GOplot (Walter et al., 2015) and ggplot2 (Wickham, 2009).

## Results

**FIGURE 1:**
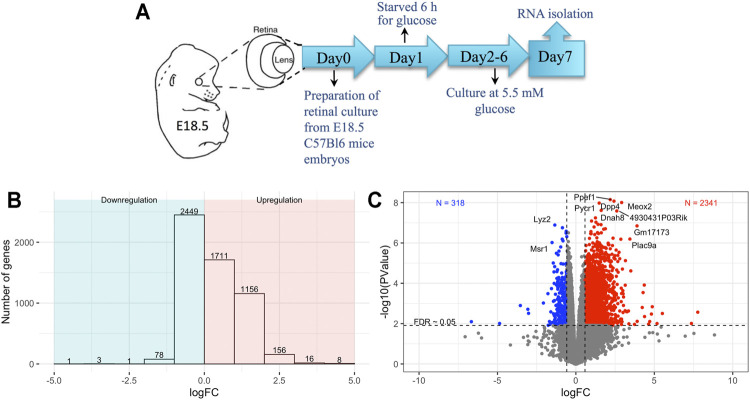
**(A)** Schematics of experimental setup, **(B)** barplot showing the number of differentially expressed genes for different log-fold-change bins, **(C)** volcano plot showing the variation of significance with log-fold-change; blue and red colors show downregulated and upregulated genes in the starved conditions with a log-fold-change greater than 50% between conditions and FDR <0.05.

Embryonic retinal cells were exposed to short-term 6-h glucose starvation to study the effects of perinatal glucose starvation on the global transcriptomic profile (*n* = 6) (Figure 1A) (Methods). Control cells were cultured under normal culture environment (*n* = 5). RNA was extracted for bulk RNA sequencing, and differential expression in the starved cells compared to controls was analyzed. A gene was labeled as differentially expressed between starved and control cells after multiple testing adjustment of *p*-values using FDR <0.05. We detected over 5,000 genes differentially expressed in response to exposure starvation; of those, 3,051 were upregulated and 2,533 were downregulated (FDR <0.05, Figure 1B and Supplementary Table S1). About 77% of upregulated genes (2,341) and 13% of downregulated genes (318) had at least a twofold change in the starved-for-glucose samples compared to non-treated controls (Figure 1C).

### Exposure to Glucose Starvation Results in Downregulation of Neuronal Retinal Marker Gene Expression

**FIGURE 2:**
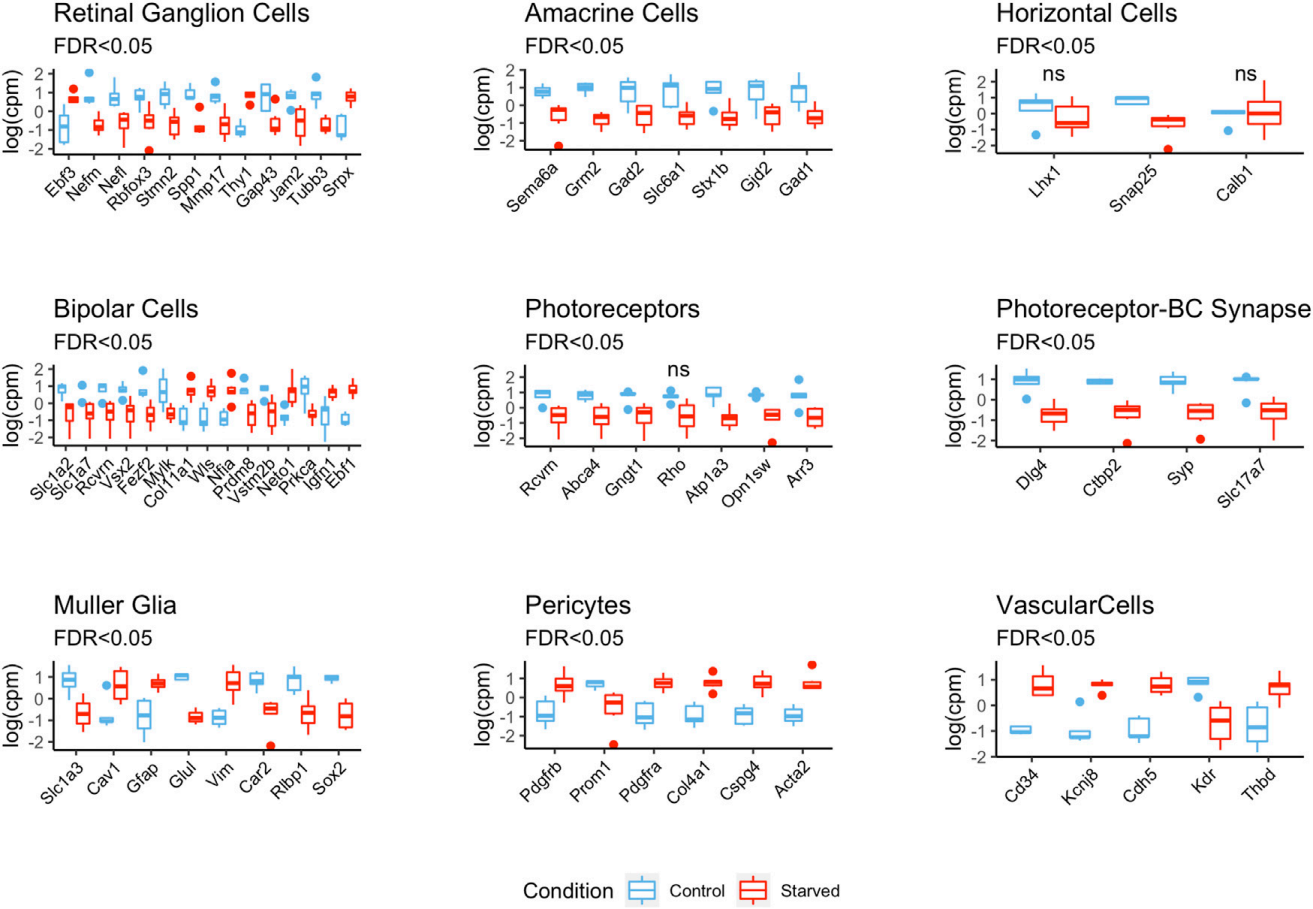
Differential expression of retinal cell markers between starved and control conditions. The expression values are centered and scaled (Supplementary Figure S4 shows unscaled plots with expression levels).

To study the effect of exposure to glucose starvation on transcriptomic profiles of different retinal cells, we analyzed expression of various retinal cell markers previously described in the literature (see Methods) (Figure 2 and Supplementary Figure S3 and Supplementary Table S2). It is worth mentioning that some of these genes were reported to be expressed in several retinal cell types and were not specific to a single cell type although they were still used as markers.

There were 23 **retinal ganglion cell (RGC)** markers detected, of which 10 (*Gap43*, *Jam2*, *Nefl*, *Nefm*, *Mmp17*, *Rbfox3*, *Spp1*, *Stmn2*, and *Tubb3*) were downregulated, while *Thy1* and *Ebf3* were highly upregulated as a result of starvation. The differential expressions of other RGC markers such as *Atoh7*, *Cartpt*, *Slc17a6*, and *Pou4f1* were not statistically significant.

Expression of **amacrine cell (AC)** markers (7 out of 16) was significantly downregulated in the starved samples. These were markers related with GABAergic cells: glutamate decarboxylase genes *Gad1* and *Gad2,* GABA transporter *Slc6a1*, and metabotropic glutamate receptor gene *Grm2*. *Sema6a*, which is an important gene for starburst ACs (SACs) and for stratification of the retinal layers (Seung and Sümbül, 2014), was also one of the downregulated AC markers.

The expression levels of horizontal cell marker genes *Lhx1*, *Snap25*, and *Calb1* were decreased in the starved cells, but only *Snap25* was found to be significantly differentially expressed with FDR <0.05.

General **bipolar cell** (**BC**) markers such as *Slc1a7*, which encode glutamate transporter; *Prdm8*; and *Vsx2* showed decreased expression after starvation exposure as well as rod BC markers *Prkca* and *Vstm2b*, which are important for BC survival and differentiation during embryogenesis. In contrast, *Col11a1*, *Ebf1*, *Igfn1*, *Neto1*, *Nfia*, and *Wls* were the upregulated BC markers probably reflecting disproportion in *On* and *Off* BC subtypes.

**Photoreceptor (PR)** marker genes *Abca4*, *Arr3*, *Atp1a3*, *Gngt1*, *Rcvrn*, and *Opn1sw* were downregulated in the starved samples, while the downregulation of rhodopsin (*Rho*) was not statistically significant. Synaptic markers such as *Ctbp2*, *Dlg4*, *Slc17a7*, and *Syp* were also highly and negatively affected by the starvation.

For the **Müller cells (MC)**, *Rlbp1*, *Glul*, *Slc1a3*, and *Car2* genes, involved in neuroglial interplay, were downregulated with starvation, while *Cav1*, *Gfap*, and *Vim* were upregulated. **Astrocyte** markers such as *Aldh1l1*, *S100b*, and *Fgfr3* were highly upregulated in the starved cells, while *Slc1a2* was downregulated. For **microglia**, *Adgre1*, *Cd40*, *Cd68*, *Aif1*, *Ptprc*, *Itgam*, and *Cx3cr1* were used as markers, and only *Cd68* was found to be differentially expressed and downregulated in the starved cells.

Notably, we have also detected a decrease in the expression levels of neuronal cell markers that belong to different lineages, such as *Sox2*, *Notch1*, and also *Pax6*—an established developmental marker of progenitor cells in the retina (Supplementary Table S3).

### Starvation Induces Proangiogenic Reprograming of the Developing Retina

Markers of **pericytes** and **vascular endothelial cells** such as *Cd34*, *Cdh5*, *Cspg4*, *Thbd*, *Pdgfrb*, and *Acta2* showed upregulation after starvation exposure, while *Vwf* and *Des* were not significantly differentially expressed. Additionally, some genes associated with vascularization such as *Tek* and *Angptl1* were also upregulated in the starved samples. VEGF genes (*Vegfa*, *Vegfc*, and *Vegfd*) were significantly upregulated after starvation exposure, but the Vegf receptor gene *Kdr* is found to be highly downregulated in the starved samples.

Given that VEGFs have been ascribed to play a pivotal role in co-patterning of nerves and vessels, we performed co-expression analysis of differentially expressed genes with *Vegfa*, *Vegfc*, and *Vegfd* in order to investigate potential VEGF-related candidates for therapeutic interrogation. Almost half of the differentially expressed genes were found to be commonly correlated across all *Vegf*s (n = 25,12, adjusted *p*-value ≤ 0.05) (Supplementary Table S5). Furthermore, to focus on vascularization-related genes in the co-expression analysis, we employed the gene list from the MGI database including many blood vessel development- and angiogenesis-related terms (Supplementary Table S6). Out of 262 differentially expressed genes related with these terms, 200 had common correlation across all three VEGF genes (adjusted *p*-value ≤ 0.05) (Supplementary Table S7).

### Increased Vascularization Was Associated With Compromised Neuronal Development in the Starved Retina

**FIGURE 3:**
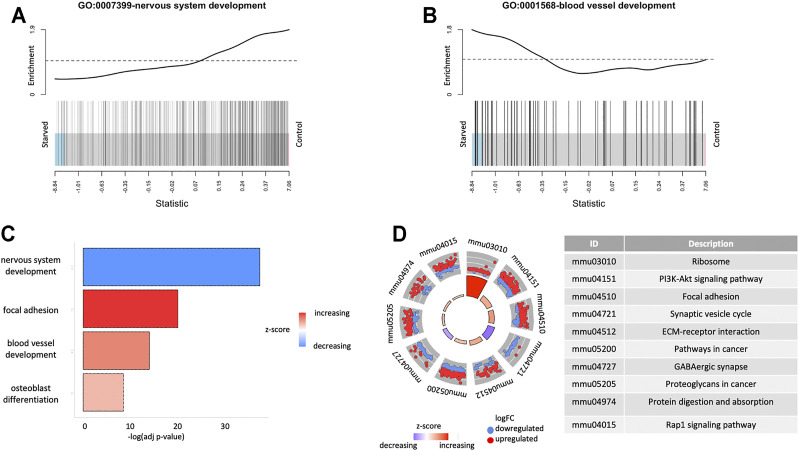
Functional enrichment results for expressed genes. **(A)** Gene Set Enrichment Analysis (GSEA) for GO terms GO: 0001568-blood vessel development and GO:0007399-nervous system development. **(B)** Overrepresentation analysis of GO terms for differentially expressed genes. **(C)** Overrepresentation analysis of KEGG pathways for differentially expressed genes.

For the investigation of the biological landscape affected by the short-term starvation exposure, we performed enrichment analysis using GO terms and KEGG pathways. Figure 3 shows specific GO biological process (BP) terms enriched for the differentially expressed genes (Supplementary Figure S4). Two of the top GO terms were nervous system development and blood vessel development, which were enriched for the downregulated (*p* = 1.4 · 10^–42^) and upregulated (*p* = 3.8 · 10^–18^) directions, respectively (Figure 3).

### Short-Term Glucose Starvation Had a Lasting Effect On Various BPs and Signaling Pathways

Figure 3D shows the KEGG pathway enrichments for the differentially expressed genes. One of the top enriched pathways is the PI3K-Akt signaling pathway (*p*_up_ = 2.6 · 10^–11^), which is among the most important signaling pathways for metabolic control, cell survival, and proliferation, demonstrating enrichment of overexpressed genes in the starved cells. Among other upregulated KEGG pathways (*p* < 10^–6^) as a result of starvation exposure were ribosome (*p* = 3.2 · 10^–30^), olfactory transduction (*p* = 7.1 · 10^–14^), focal adhesion (*p* = 1.4 · 10^–10^), ECM–receptor interaction (*p* = 7.0 · 10^–10^), and those related to cancer (*p* = 6.3 · 10^–8^). On the contrary, synaptic vesicle cycle (*p* = 6.5 · 10^–13^) and GABAergic synapse (*p* = 5.6 · 10^–9^) pathways were downregulated, which can imply altering of neuroglial relations and synaptic transduction in the retina after glucose deprivation.

### Genes Regulating Fatty Acid Elongation Were Upregulated After Glucose Starvation to Compensate for Reduced Expression of Glycolytic Enzymes

**FIGURE 4:**
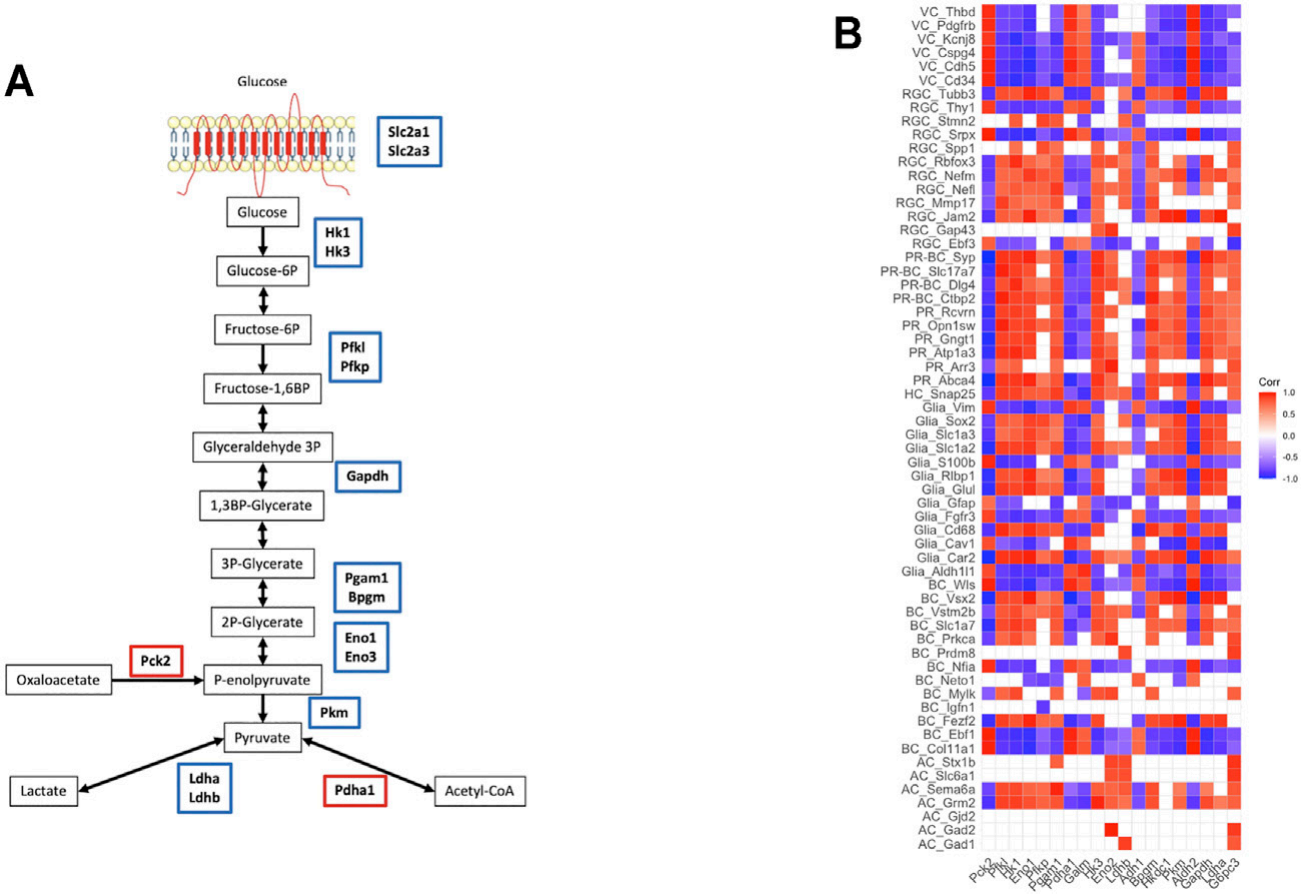
Metabolic changes after starvation exposure: **(A)** glycolysis pathway—the genes encoding the enzymes functioning in the pathway were downregulated, **(B)** the correlation between retinal cell markers and glycolysis/gluconeogenesis genes (VC: vascular cells, RGC: retinal ganglion cells, PR-BC: photoreceptor-bipolar cell synapses, PR: photoreceptor cells, HC: horizontal cells, BC: bipolar cells, AC: amacrine cells).

To shed light on the processes involved in energy balance affected by exposure to starvation, we analyzed genes involved in glucose, lipid, and amino acid metabolism. As expected in the condition of starvation for glucose, there was decreased expression of the genes involved in several steps of glycolysis including glucose transport (Figure 4A). These genes encode enzymes in the pathway from glucose to pyruvate metabolism, as well as *Ldha* and *Ldhb* encoding for l-lactate dehydrogenase, which catalyzes pyruvate-to-lactate conversion (Kanehisa and Goto, 2000; Slenter et al., 2018). In line with decreased expression of glycolytic genes, glucose transporters such as *Slc2a1* (GLUT1) and *Slc2a3* (GLUT3) were downregulated after starvation (FDR <0.05).

On the contrary, genes encoding for enzymes in the fatty-acid elongation pathway in the endoplasmic reticulum such as *Elovl1*, *Elovl3*, *Elovl5*, *Fads1*, *Scd1*, *Hacd4*, *Acot1*, and *Acot2* were highly upregulated in the starved samples indicative of increased biosynthesis of fatty acids. Similarly, genes involved in amino acid transport and metabolism such as *Dpp4*, *Xpnpep1*, *Xpnpep2*, *Mme*, and *Slc1a5* are highly upregulated in the starved cells (Supplementary Table S1).

Finally, to investigate the glycolytic and metabolic states of different retinal cell types, we assessed correlations of neuronal markers with glycolysis genes (Figure 4B). Assessment of the links between metabolic characteristics and cell-specific markers demonstrated a positive correlation between expressions of *Ldha* and PR-specific genes (*Abca4*, *Arr3*, *Atp1a3*, *Gngt1*, and *Rcvrn*) involved in different aspects of PR functioning including all-trans-retinal aldehyde and cation transport, regulation of rhodopsin activity, proper propagation, and termination of signaling. Moreover, glycolytic enzymes and *Ldha* repression were associated with downregulation of signaling molecules involved in neuronal subtype-specific patterning and axonal growth, as well as in synaptic plasticity regulation. Downregulation of *Hk1*, *Hk3*, *Hkdc1*, *Pfk*, and *Pkm* positively correlated with lower expression of genes encoding adhesion molecules and intercellular junctions (*Jam2* and *Mmp17*), proteins regulating neuronal differentiation (*Rbfox3*) and growth (*Stmn2*), axonal transport (*Nefl*, *Nefm*, and *Tubb3*), and neuroprotection (*Spp1*) in RGC. Glycolytic enzymes and *Ldha* expression were in synchrony with downregulation of glutamate and GABA signaling and transport in both neurons and glial cells. Additionally, glycolytic enzyme expressions showed profound negative correlation with vascular marker expressions.

## Discussion

The main findings in the present study provide evidence of differential changes in expression of genes contributing to retinal development and functioning after short-term metabolic disruption. Obtained data allow us to generate a hypothesis of irreversible and detrimental reprogramming of neurovascular unit (RNVU) formation during retinal development after early-life exposure to glucose starvation. Even after a “relief” period of normal conditions, the effects on the transcriptomic landscape of the retina were still immense. The key features appear to include multiple cellular pathways highlighting disrupted gene expression in the retina. Specifically, transcriptomic analysis has revealed a profound decrease in the expression of neuronal markers, while genes encoding for vascular markers were upregulated, similar to the diabetes-associated increase of angiogenesis in diabetic retinopathy.

Investigation of the molecular mechanisms underlying retinogenesis is important in order to gain knowledge on how to enlighten the path ahead for prevention or treatment of retinopathy in adults with diabetes. The top differentially expressed genes affected by starvation comprised *Ppef1*, which encodes for the protein product suggested to play a role in sensory neuron function and development; *Dpp4*, encoding a protease enzyme involved in the cleavage of a broad range of vasoactive peptides, which is also an established drug target for type 2 diabetes (Dicker, 2011); *Meox2* gene, known to regulate angiogenesis and myogenesis; and *Pycr1* and *Dnah8*, involved in the ATP-related processes. These findings provide further support for involvement of multiple mechanisms including neuronal, vascular, and energy metabolism that together may contribute to compromise early development of the entire RNVU.

A major pathological feature of advanced and severe forms of retinopathy in patients with diabetes is characterized by accelerated proliferative angiogenesis. This gives rise to an increased growth of small and immature vessels in the retina, which are susceptible to breakage and bleeding. In the present experiments using embryonic retinal cells, the vascular cell markers showed overall upregulation after exposure to glucose starvation, which could be a consequence of lack of necessary nutrition. The restructure of the vascular network might therefore be needed to reach resources even after a short-term glucose deprivation. In support of this, expression of other genes important for blood vessel development (*Tek* and *Angptl1*) was also upregulated along with the retinal vascular markers. As was previously reported, starvation causes acute energy depletion that can create a hypoxia-like condition (Rego et al., 1998). As a consequence of this, triggering of mechanisms to promote increased blood supply takes place, typically involving activation of HIF—a major driver for the transcription of *VEGF*s and overall over 60 genes adjusting cells to a hypoxic state (Sapieha et al., 2010). In this study, we have demonstrated that all *VEGF* genes (*Vegfa*, *Vegfc*, and *Vegfd*) were significantly upregulated after starvation exposure. Notably, elevated *VEGF-C* and *VEGF-D* levels were found in the retinal pigment epithelium (RPE) of patients with age-related macular degeneration (Vellanki et al., 2016). There are also data confirming a significant role of *VEGF-D* in retinal angiogenesis and ganglion cell protection under excitotoxic injury (Schlüter et al., 2020). In addition, it was demonstrated that hypoxia-induced expression of *VEGF-C* in the retina is as potent as *VEGF-A* in inducing pathological retinal neovascularization in PDR and retinopathy of prematurity (Campochiaro, 2015; Singh et al., 2015; Vellanki et al., 2016). In the present study, positive correlations between *Vegf*’s expression and vascular markers indicate strong promotion of angiogenesis.

*Kdr* (or *Vegfr2*) that binds *Vegfa*, *Vegfc*, and *Vegfd* in the retina was strongly downregulated in starved samples. In contrast to *Flt1* (*Vegfr1*) that is restricted to endothelial cells, *Kdr* is abundantly expressed in the neuroretina (Penn et al., 2008). Importantly, during retinal neurogenesis, *Kdr* is also expressed by neural progenitor cells and retinal neurons (Hashimoto et al., 2006). High *Kdr* expression in embryonic retinal nerve cells under physiological conditions titrates VEGF to moderate spatial patterning of angiogenesis and limits internal retinal vascularization. It was shown that loss of *Kdr* in neurons caused misdirected angiogenesis toward neurons, resulting in abnormally increased vascular density around neurons (Okabe et al., 2014). Interestingly, Müller cell survival and proliferation during retinal development depend on VEGFR- and MAPK-related signaling (Liu et al., 2019). Mice with conditional knockout of *Kdr* demonstrated significant loss of Müller cells under diabetes/hypoxia, which accelerated retinal degeneration. These show the critical role of VEGF signaling in glial cells’ viability and neuronal integrity (Fu et al., 2018). Thus, increased VEGF expression after glucose deprivation may reflect activation of proangiogenic activity of retinal glial cells and provokes abnormal angiogenesis in the inner retinal compartment, whereas downregulation of *Kdr* can be related with reduced neurogenesis. A simultaneous increase of vascular markers supports this idea. The fact of increased expression of VEGF and other vascular markers reflects strengthening of the glial–vascular relationship, while neuroglial relations were compromised.

The key master regulator of metabolic relationships between different retinal cells in RNVU is macroglia (Müller cells and astrocytes). Müller cells are the main contributors of glutamate and GABA recycling in the retina and responsible for glutamate uptake from the synaptic cleft to prevent neurotoxicity (Bringmann et al., 2013). As glutamate and GABA are principal neurotransmitters ensuring the radial and lateral synaptic pathways, respectively (Yang, 2004), their downregulation could lead to the decrease of the retinal functional integration and contribute to neuronal degeneration (Bringmann et al., 2013). Similar to GABA, observed downregulation of *Glul* may reflect impaired regulatory function of Müller cells in neurotransmission and weakening of the neuroglial relationship. On the other hand, this may be related with reactive glial cells (de Melo et al., 2012) and the breakdown of the blood–retinal barrier (Shen et al., 2009), considering detected upregulation of *Gfap* and *Vim*. Reactive glia in turn may contribute to neuronal death via glutamate excitotoxicity (Sundstrom et al., 2018).

Notably, decreases in expression of some genes such as *Sema6a*, which has an important function in stratification of retinal layers (Seung and Sümbül, 2014), may furthermore indicate the overall dissociating effect of starvation exposure on the retinal structure. We observed a strong decrease in expression of marker genes for photoreceptors and photoreceptor-to-bipolar cell synapses, indicating that these cells and their connections are among the most vulnerable to abnormal metabolic conditions. Concomitant downregulation of other neuronal markers, especially the markers for progenitors and RGCs, and the upregulation of vascular and reactive glial markers can suggest that the short-term starvation for glucose may not only cause a retardation in the temporal development but might also give rise to the events promoting future neurodegeneration.

Interestingly, the expression of glucose transporters and glycolytic enzyme genes after exposure to glucose starvation correlated positively with photoreceptors and RGC, while it negatively correlated with VEGF expression in the retina. The similar changes were revealed under retinopathy of prematurity. Increased angiogenesis was associated with decreased retinal *Glut1* and glycolytic enzyme expressions. In line with this, it was demonstrated that improvement of glucose uptake and glycolysis may restore retinal neuron formation and normalize retinal angiogenesis (Han et al., 2019).

When interpreting reciprocal changes in vascular drivers and glycolytic enzyme expression, it is important to underline the spatial distribution of distinct expressions and various responses of different retinal cells to hypoxia. The main site of aerobic glycolysis enzyme expression and lactate production is PRs cells located in the outer retina, whereas the main source of VEGF is Müller and ganglionic cells located in the inner retina. Within the retina, PRs rank among the highest-energy-consuming systems. Although PRs are rich in mitochondria and oxidative phosphorylation (OXPHOS) enzymes, these cells convert most of their glucose to lactate through aerobic glycolysis—a process known as the Warburg effect and accounts for about 80–90% of glucose metabolism in adult PRs (Chinchore et al., 2017; Narayan et al., 2017). It is speculated that aerobic glycolysis provides sufficient glucose influx to the pentose-phosphate pathway (PPP) with the subsequent generation of NAPH and lipid synthesis, allowing the recycle of the appropriate amounts of visual pigment (Sun et al., 2008). Lactate production depends on the expression and activity of LDHA, the enzyme critical for the Warburg effect, which was also downregulated in the retina due to starvation. The lack of LDH-A expression was detected in rats with inherited “retinitis pigmentosa,” associated with pathological loss of the photoreceptors (Narayan et al., 2019). This finding supports the notion that the PRs are particularly susceptible to inhibition of glycolysis. Lactate production by PRs is also important for the retinal glial cell functioning. In contrast to PRs, Müller glial cells do not express hexokinase or any pyruvate kinase isoform required for glycolysis (Lindsay et al., 2014; Rueda et al., 2016). Lactate provided by aerobic glycolysis in PRs is used as a fuel by Müller cells and RPE. Additionally, lactate, rather than glucose, is the most effective source of carbon for glutamine synthesis by Müller cells (Gardner and Davila, 2017). Naturally, downregulation of glycolytic enzymes, such as *Hk1/3* and *Impdh*, in the starved retina was associated with a decline in expression of other molecules involved in glutamate turnover, neuronal differentiation, axonal growth, and synaptic transduction. This highlights the importance of a tight interface between retinal neurons and Müller cells that work together as an ecosystem to build metabolically specialized and interdependent RNVUs.

Importantly, the KEGG pathway analysis demonstrated upregulation of gene clusters related to PI3K-Akt and cancer-associated pathways in addition to the regulation of cell-matrix interplay, cell adhesion and migration, protein synthesis, and proteolysis (Figure 3D). *Akt* was found to be increased as a result of hyperglycemia, which is regarded as the primary cause of the development of retinopathy in the patients with diabetes (Qin et al., 2015). The increase in the signaling through PI3K promotes fibrosis in the retina, which in turn aggravates development of retinopathy. In support of activation of fibrosis in the samples starved for glucose, the extracellular matrix genes such as collagens (such as *Col4a1* and *Col1a1*), fibronectin (*Fn1*), and laminins (such as *Lamb1* and *Lama4*) were significantly differentially expressed with high fold changes. This signaling pathway may be a target for therapeutic intervention of pathogenic angiogenesis similar to those being developed in cancer (Sasore et al., 2014).

### Limitations

The global expression analyses in the present study are performed using mRNA sequencing, and the corresponding protein data and morphological evidence are lacking. Therefore, additional experiments would be needed to expand current results for further validation at the protein expression level. Nevertheless, our observations on downregulation of glycolytic enzymes and their strong correlation predominantly with PR markers are in support of the suggested critical importance of main regulators of aerobic glycolysis in PR not only in the enzymatic reactions but also possibly in acting as neuroprotective mechanisms critical in maintaining PR health, viability, and survival (Weh et al., 2020). It is important to note that current results were generated using mouse embryonic retinal cells, and validation in humans would be warranted. To this extent, a genome-wide association study for severe retinopathy is ongoing in our cohort of Ukrainian patients with type 2 diabetes who were exposed to famine at birth, as a part of the National Ukrainian Registry (Fedotkina et al., 2021).

In summary, the present data provide hypothesis-generating evidence that perinatal glucose deprivation may cause metabolic adaptations in different compartments of the embryonic retina, leading to alterations in the developmental program of the entire RNVU. As was shown in the present study by experimental modeling of starvation for glucose of embryonic retinal cells, early-life metabolic adaptations might trigger reprogramming of retinogenesis with alteration of retinal neurogenesis towards abnormal angiogenesis. Thus, a negative correlation between the expression of glycolytic enzymes and VEGFs in the retina after starvation may reflect the desynchronization of neuroglial interplay and dissociation between outer and inner retinal functions and therefore choroid and retinal vasculature formation. These findings highlight the crucial importance of restoring the balance between neuroglial and glio-vascular function. A combined strategy including antiangiogenic drugs and metabolic correction could be proposed as a beneficial therapeutic approach. The latter could include governed stimulation of aerobic glycolysis and activation of the glutamate transporters to improve neuroglial coupling through lactate production supporting metabolic processes, neurotransmitter exchange, and synaptic transduction.

## Data Availability

The datasets presented in this study can be found in online repositories. The names of the repository/repositories and accession number(s) can be found below: Experiment ArrayExpress accession: E-MTAB-10705.
