# Supplementary material for: Starvation to Glucose Reprograms Development of Neurovascular Unit in Embryonic Retinal Cells
Source: Front Cell Dev Biol. 2021 Nov 18;9:726852. doi: 10.3389/fcell.2021.726852 (PMC8636675; doi:10.3389/fcell.2021.726852)
Supplement: Supplementary file 1 [file DataSheet1.PDF]

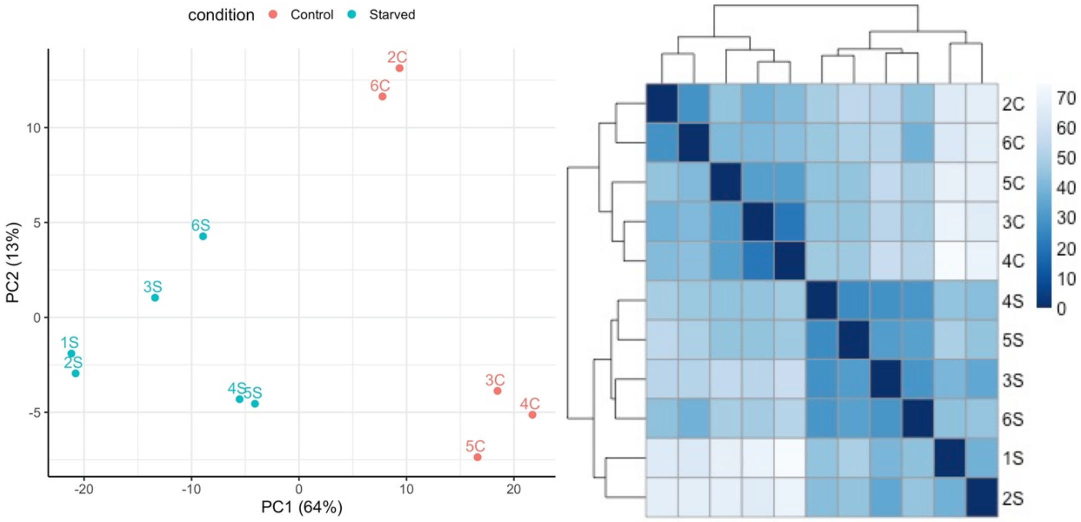

Figure S1 – a) PCA plot of samples, b) hierarchical clustering between samples. The samples are separated in the first dimension on the PCA biplot and hierarchically clustered in 2 main branches according to their conditions.

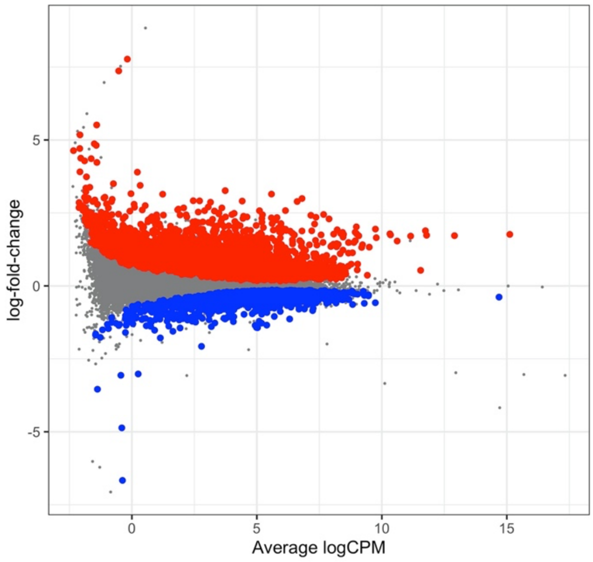

Figure S2 – The variation of log-fold-change and average expression in terms of logCPM. Blue and red colors show down and upregulated genes in the starved conditions with log-fold-change greater than 50% between conditions and FDR < 0.05

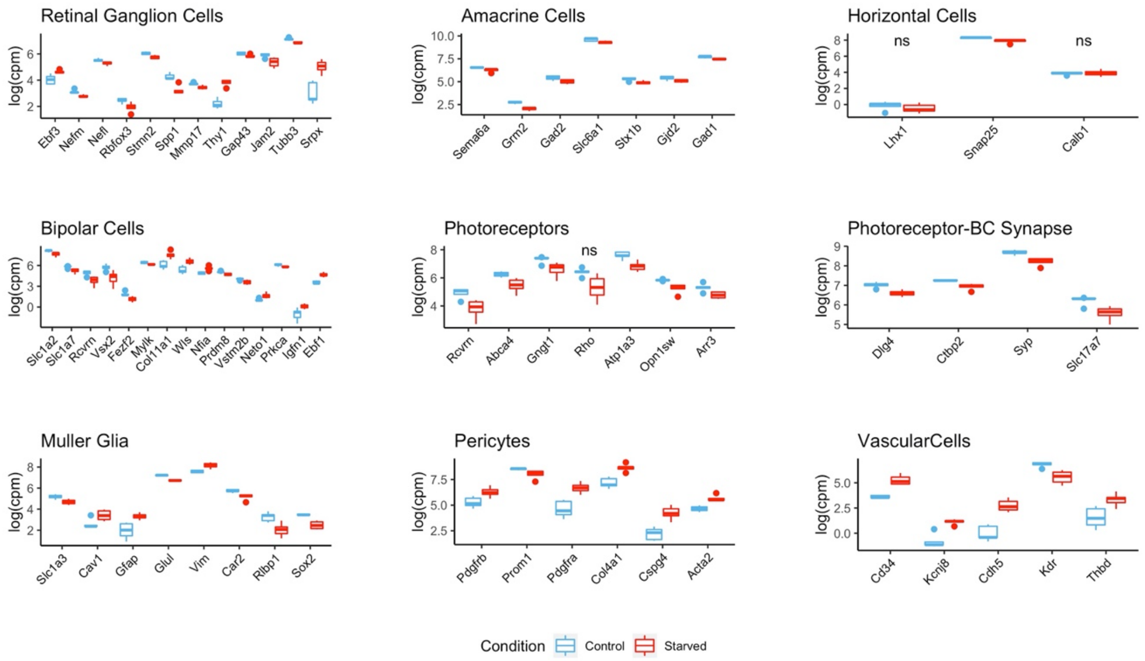

Figure S3 – Differential expression of retinal cell markers between starved and control conditions.

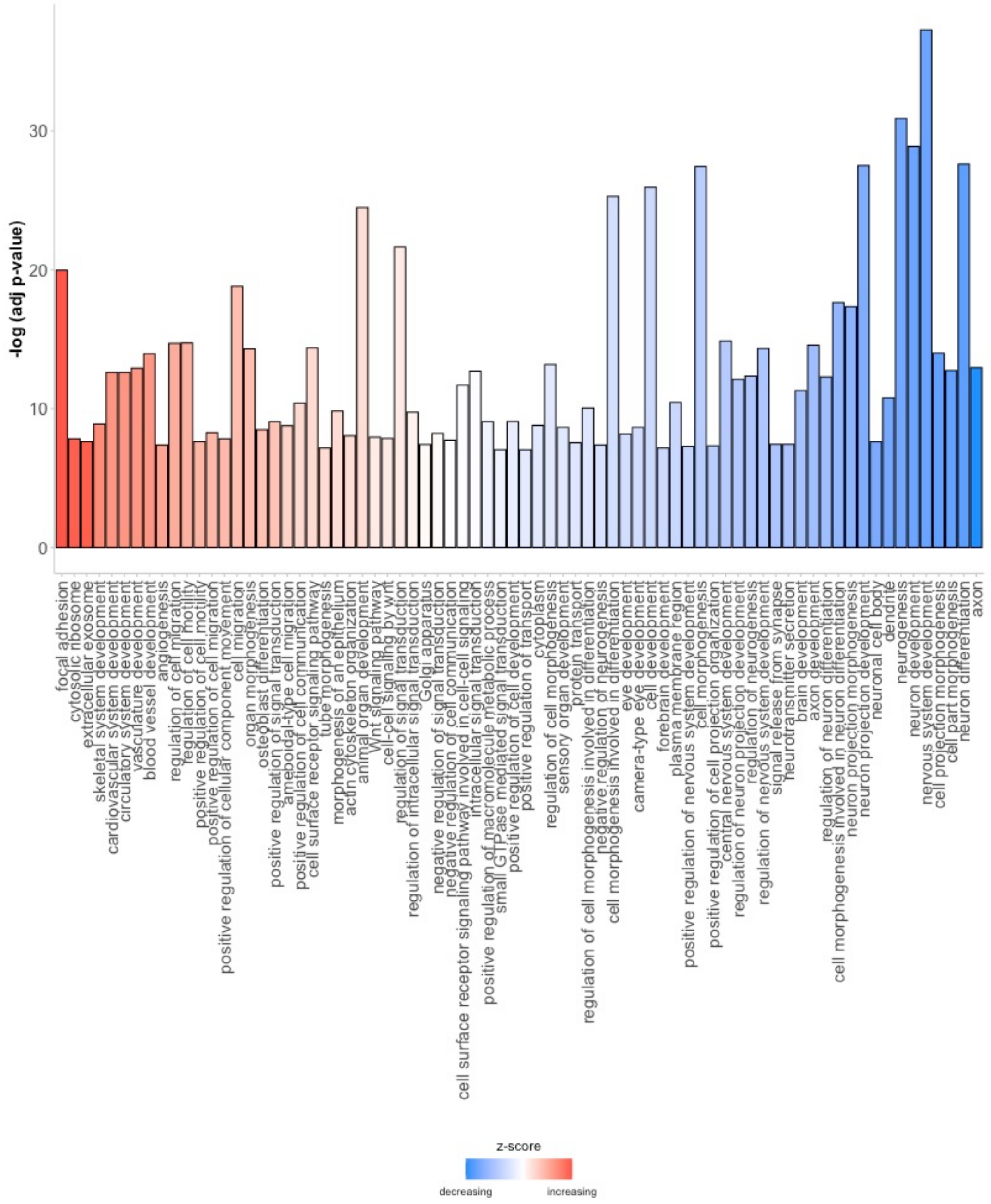

Figure S4 – Gene ontology enrichment of differentially expressed genes – enlarged plot for top enriched terms

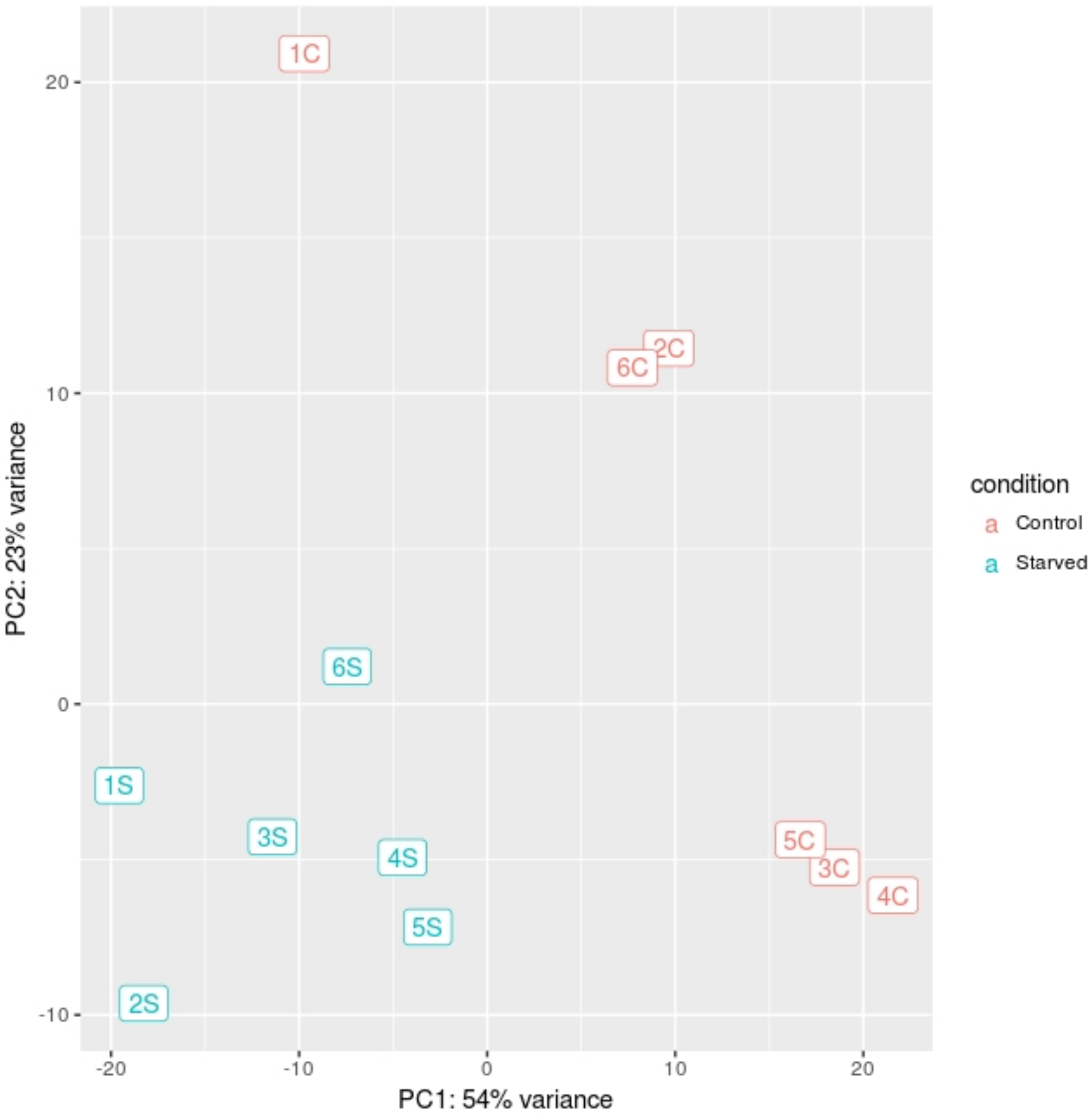

Figure S5 – PCA biplot before removal of outlier sample (1C)
